# Case Report: Multimodality imaging of a bronchogenic cyst in the interatrial septum

**DOI:** 10.3389/fcvm.2024.1466016

**Published:** 2024-10-09

**Authors:** Ma Mingming, Zhao Yana, Chen Ran, Zhu Qingqing, Zhao Bowen

**Affiliations:** ^1^ Department of Diagnostic Ultrasound & Echocardiography, Sir Run Run Shaw Hospital, Zhejiang University College of Medicine, Technical Guidance Center for Fetal Echocardiography of Zhejiang Province and Sir Run Run Shaw Institute of Clinical Medicine of Zhejiang University, Hangzhou, China; ^2^ Department of ECG, Sir Run Run Shaw Hospital, Sir Run Run Shaw Institute of Clinical Medicine of Zhejiang University, Hangzhou, China; ^3^ Department of Radiology, Sir Run Run Shaw Hospital, Sir Run Run Shaw Institute of Clinical Medicine of Zhejiang University, Hangzhou, China

**Keywords:** congenital anomaly, bronchogenic cyst, interatrial septum, diagnosis, therapy

## Abstract

Intracardiac bronchogenic cysts (IBCs) are very rare. To date, only a few cases of IBC have been reported in the literature. We report a case of a bronchogenic cyst that arose from the interatrial septum in a 42-year-old man who presented with symptoms of palpitation. A unilocular cystic lesion of the heart was found initially on echocardiography and subsequently on computed tomography and magnetic resonance imaging. The diagnosis was further confirmed by histopathology after surgical resection. Multimodality imaging played a crucial role in the diagnosis and treatment of such rare lesions.

## Background

A bronchogenic cyst is considered a rare congenital lesion that originates from the ventral foregut during embryogenesis (1, 2). It can usually be found in the mediastinum or lung (3). Intracardiac bronchogenic cysts (IBCs) are very rare, accounting for only 1.3% of all primary cardiac and pericardial tumors. The majority of bronchogenic cysts are either asymptomatic and discovered incidentally through imaging or they present with symptoms caused by the compression of surrounding structures, such as dyspnea, atrioventricular block, or superior vena cava syndrome. This case was assessed using multimodal imaging and underwent successful resection.

## Case presentation

**Figure 1:**
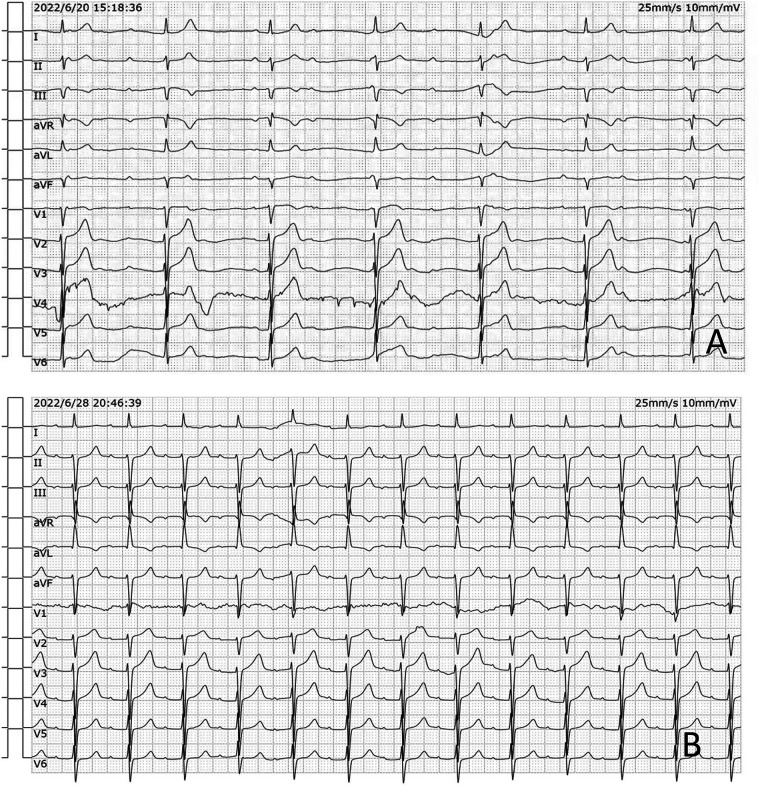
**(A)** The preoperative 12-lead ECG showed a third-degree atrioventricular block. **(B)** The postoperative ECG showed a first-degree atrioventricular block.

**Figure 2:**
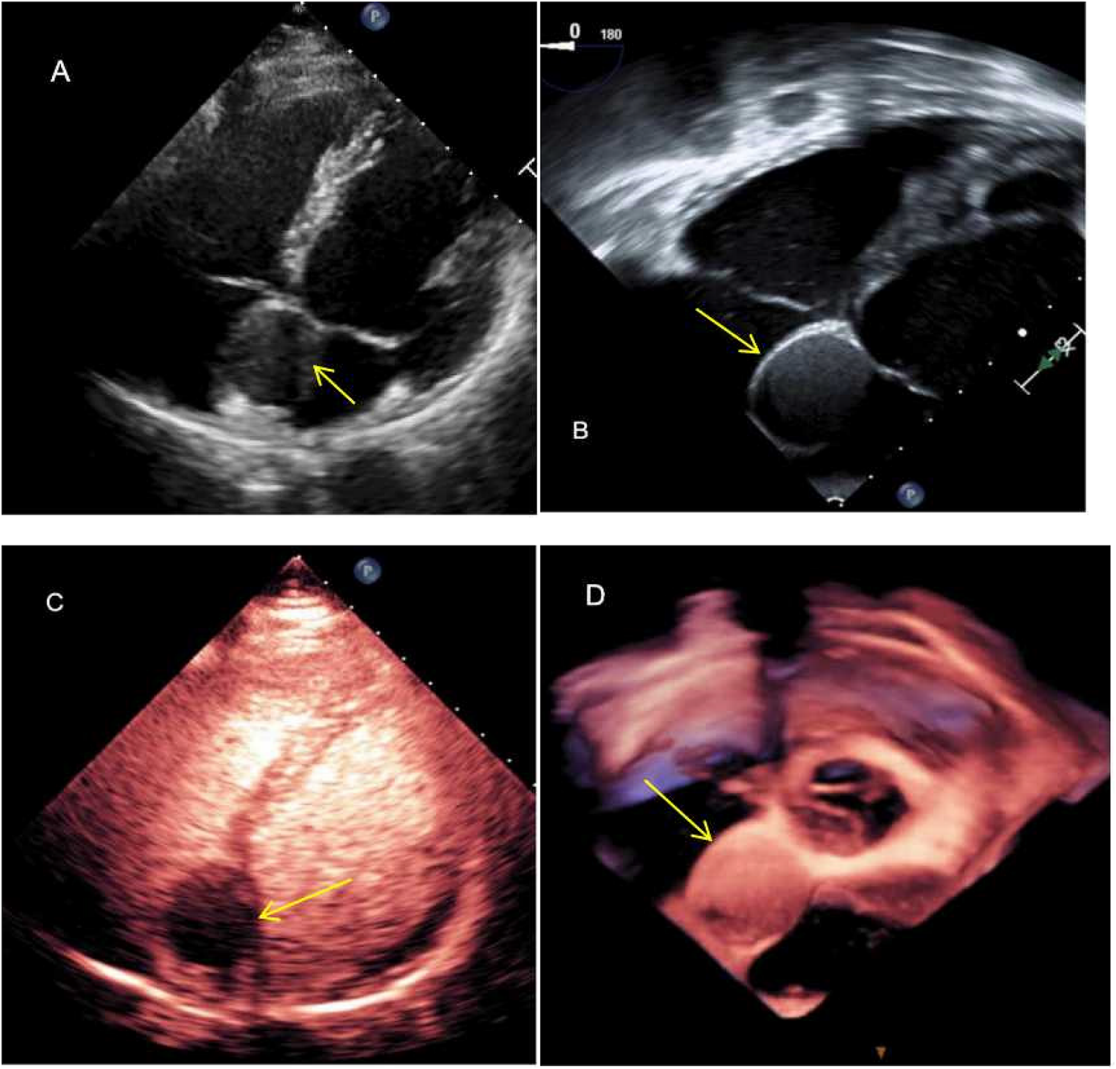
Echocardiography showing the well-defined ovoid interatrial lesion (arrow). **(A)** Transthoracic echocardiography showing a well-demarcated hypoechoic ovoid mass embedded in the interatrial septum in the apical four-chamber view; **(B)** TEE showing a homogeneous, isoechoic mass at the interatrial septum—the mass is well-defined and has a smooth surface; **(C)** the mass was not enhanced by the use of a contrast agent; and **(D)** three-dimensional TEE illustrating the relationship between the mass and surrounding structures.

**Figure 3:**
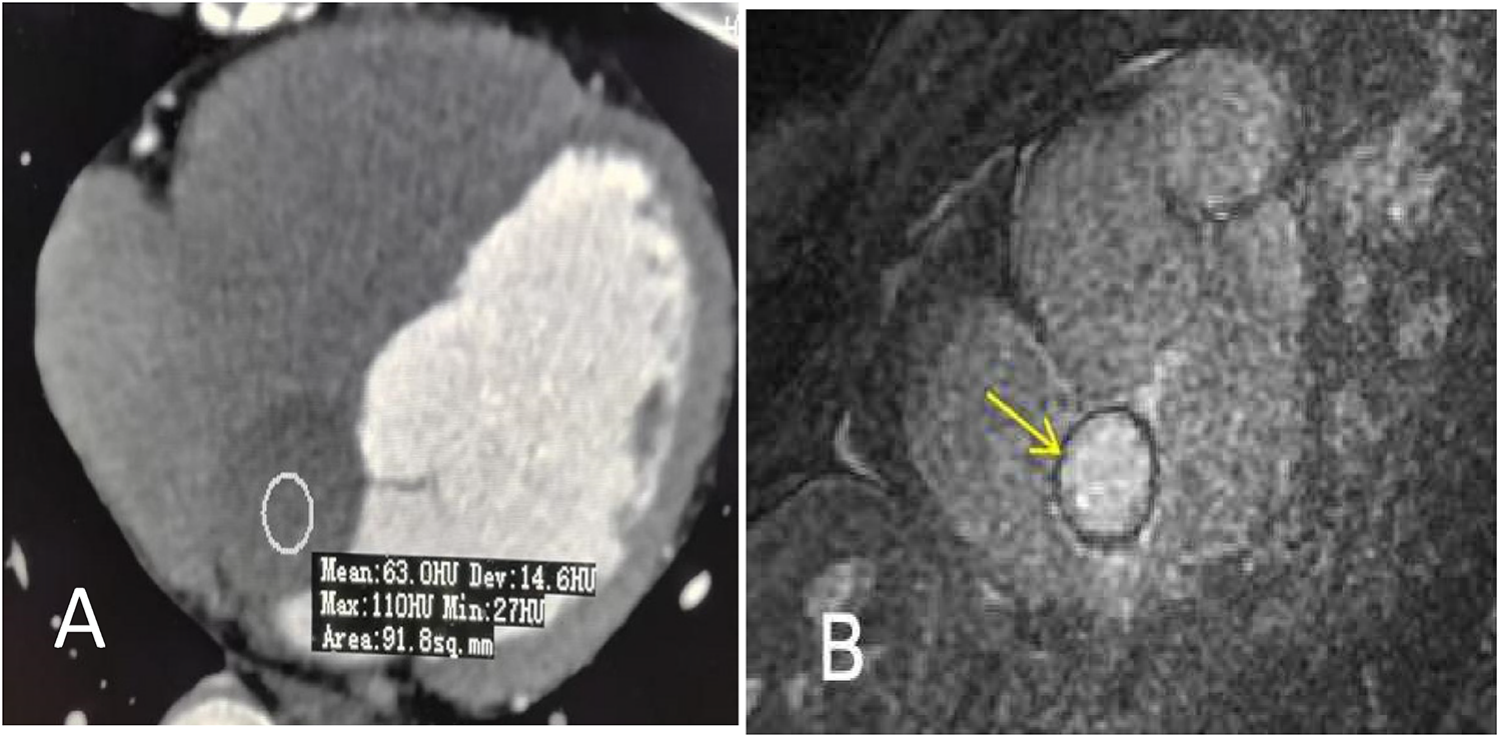
**(A)** Contrast-enhanced CT shows a low-intensity ovoid nodule without obvious enhancement at the interatrial septum (arrow). The mean attenuation value was approximately 63 Hu. **(B)** Cardiac MRI shows a round mass in the interatrial septum measuring 30 mm × 20 mm (arrow). Neither fat deposition nor late gadolinium enhancement were identified in the tumor.

A 47-year-old man presented to a local clinic with complaints of palpitation. Physical examination and chest radiography were unremarkable; however, a 12-lead electrocardiogram (ECG) showed a third-degree atrioventricular block (Figure 1A). Transthoracic echocardiography (TTE) revealed normal ventricular and valve function; however, a well-demarcated hypoechoic ovoid mass measuring 29.9 mm × 25.6 mm was identified in the interatrial septum (IAS) (Figure 2A). In addition, transesophageal echocardiography (TEE) revealed a large, homogeneous, hypoechoic mass in the near field on mid-esophageal views (Figure 2B). The mass had no stalk, and color Doppler echocardiography revealed no color flow signal within the mass. Myocardial contrast echocardiography (MCE) revealed a hypoechogenic mass in the atrial septum, with clear and regular boundaries and no obvious contrast agent perfusion (Figure 2C). Three-dimensional TEE demonstrated the relationship between the mass and surrounding structures (Figure 2D). Subsequent computed tomography (CT) (SOMATOM Definition Flash) coronary angiography revealed no obvious stenosis in the left main coronary artery, anterior descending branch, circumflex branch, or right coronary arteries. A low-density ovoid mass was found in the atrial septum, with well-defined borders, no significant enhancement, and an absence of feeding vessels from the coronary arteries (Figure 3A). Magnetic resonance imaging (MRI) (SIGNA EXCITE HD 1.5 T) identified a mass in the atrioventricular septal region (Figure 3B). Neither fat deposition nor late gadolinium enhancement were observed in the tumor. Based on multimodality imaging, the mass was most likely consistent with a cyst.

**Figure 4:**
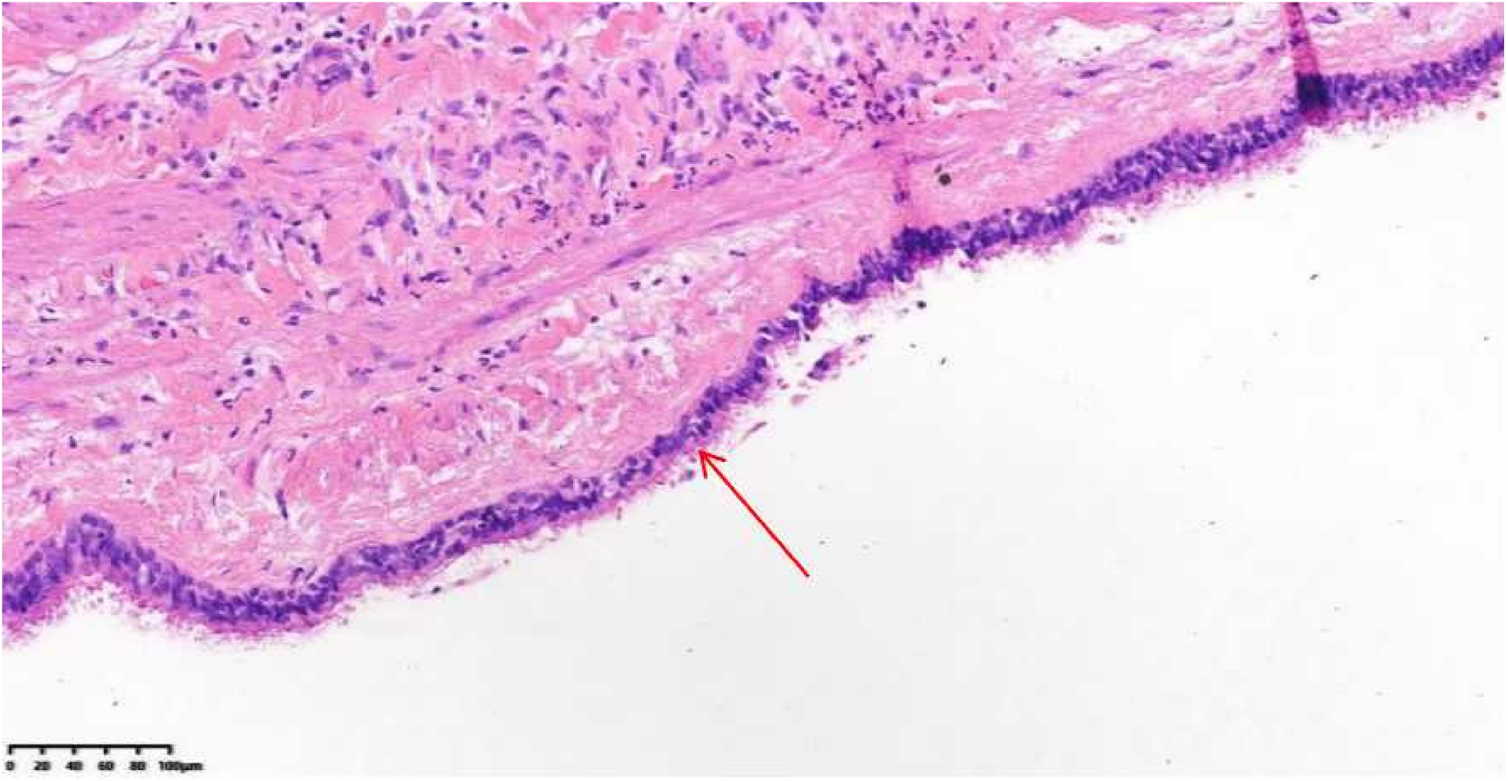
Histology shows the surface of the cystic lesion lined by pseudostratified ciliated columnar epithelium (arrow), smooth muscle cells within the fibrous tissue wall of the cyst, and acute inflammatory cells.

Given the above information, surgical resection of the cystic tumor was planned via median sternotomy. The position of the heart and great vessels was normal. A round mass measuring approximately 40 mm × 30 mm, with a smooth surface, was identified in the middle of the atrial septum and was completely resected. The mass was a cyst containing muddy, milky mucous liquid. Histology revealed that the cyst wall was composed of stratified ciliated columnar epithelium, with smooth muscle cells also observed within the wall. The final diagnosis, based on histopathologic examination, was a bronchogenic cyst (Figure 4). The patient's recovery was uneventful. The postoperative ECG showed a first-degree atrioventricular block (Figure 1B). The patient was asymptomatic without recurrence during the 2 years of follow-up.

## Discussion

The occurrence of cardiac masses is rare. They are mostly diagnosed as an incidental finding during various imaging examinations. Echocardiography is typically the first-line diagnostic technique used to evaluate patients with suspected cardiac tumors as it is widely available and free of ionizing radiation (4). However, echocardiography is limited owing to its dependence on the echogenic window and its limited ability for tissue characterization. MRI has been proven to be superior to echocardiography owing to its excellent soft tissue contrast, high temporal and spatial resolution, and lack of ionizing radiation (5). Thus, the diagnostic and therapeutic planning for patients with suspected cardiac tumors is primarily dependent on multimodal imaging tools.

When evaluating a cardiac mass, several differential diagnoses need to be considered, such as cardiac thrombus, cardiac tumor, and normal anatomical variants. Cardiac thrombi are more commonly seen in patients with a reduced left ventricular (LV) ejection fraction, and their presence is often associated with a lack of first pass prussian and late gadolinium enhancement (LGE). Cardiac tumors are rare and are typically classified as benign tumors or malignant tumors. Malignant tumors tend to be larger, have a predilection for the right heart, are more often infiltrative, and are more likely to be isointense on T1 black-blood weighted images. They also exhibit higher proportions of LGE (6). Three-quarters of cardiac tumors are benign. The most common types of benign tumors are myxomas (42%), papillary fibroelastomas (23%), and cysts (22%). Myxomas present themselves as mostly mobile masses attached to the left atrial side of the IAS, are heterogeneous, perfused, and show LGE. Papillary fibroelastoma, which rarely occurs, is typically pedunculated and frequently involves the cardiac valves. However, none of these show any enhancement after gadolinium injection. For intracardiac cysts, echinococcal cysts, congenital blood cysts, and bronchogenic cysts should be included in the list of possible diagnoses. The diagnosis of echinococcal cysts requires a history of travel to endemic areas or a medical history of a visceral hydatid cyst. Echinococcal cysts frequently involve the left ventricular myocardial wall and interventricular septum. Specific imaging features include calcification of the cyst wall, the presence of daughter cysts, and membrane detachment. Congenital blood cysts, which are most commonly observed in infants, are typically located along the lines of closure of the valvular endocardium.

IBCs were first described by Jöel in 1890 during an autopsy. However, the first surgical removal of an interatrial bronchogenic cyst was reported by Soeda et al. a century later, in 1996 (7). To the best of our knowledge, approximately 30 cases of intrapericardial bronchogenic cysts have been published in the literature. We reviewed the relevant studies and summarized our findings in Table 1.

**Table 1:** Characteristics of case reports of the intracardiac bronchogenic cysts in the literature.

Reference	Year	Age	Gender	Location	Max. dimension mm	Contents	Symptoms	Complication
Shimizu et al. (8)	1990	36	F	Intrapericardial	60	Unilocular cystic, milky white gelatinous	Chest pain, cough, low-grade fever	NA
Soeda et al. (7)	1996	43	F	IAS	NA	NA	Asymptomatic	NA
Kawase et al. (9)	2002	39	M	IAS	26	Creamy, white-yellow colored liquid	Left back pain	NA
Kobza et al. (10)	2003	34	F	Intrapericardial	60	NA	Shortness of breath and chest pain	NA
Prates et al. (11)	2003	48	F	RV	42	NA	Dyspnea	Right bundle branch block
Lee et al. (12)	2005	68	F	LA	35	Unilocular, tan jellylike, seromucinous	Dyspnea	Atrial fibrillation, PLSVC
Weinrich et al. (13)	2005	73	M	RV	10	A colorless, thread-forming fluid	Dyspnea	NA
Chen (14)	2006	70	M	IAS	30	Sticky milky white, mucus	Intermittent brief tachycardia	Atrial fibrillation, PLSVC
Wei et al. (15)	2006	2	M	LV	20	Thin-walled multiple cysts	Intermittent precordial pain	NA
Inzani et al. (16)	2006	5	F	The pars membranacea septi	10	Mucus	Premature, ventricular complex	NA
Klass et al. (17)	2007	42	F	LV	30	White-yellow mucous	Atypical angina	NA
Azeem et al. (18)	2008	47	F	Transverse sinus	40	NA	Acute chest pain	NA
Martinez-Mateo et al. (19)	2008	43	M	IAS	25	White and dense fluid	Syncopal	Third-degree atrioventricular block
Borges et al. (20)	2009	43	F	IAS	44	NA	Incidentally detected	Atrioventricular block
Vaideeswar et al. (1)	2011	6 m	F	IAS	10	White colored mucinous	Failure to thrive, recurrent upper respiratory tract infection	VSD
Jiang et al. (21)	2013	36	F	IAS	32	Yellow, jellylike fluid	Palpitation and mild dyspnea	ASD
Jiang et al. (21)	2013	29	F	IAS	18	Yellow, jellylike, fluid	Palpitation and dyspnea	ASD
Park et al. (22)	2014	35	M	IAS	21	Amber-colored mucous fluid	Incidentally detected	First-degree atrioventricular block
Forcillo et al. (23)	2015	41	F	IVS	15	Cloudy liquid with white aggregates	Heart murmur	NA
Wang et al. (24)	2016	41	M	LV	25	White mucous fluid	Intermittent precordial, pain	NA
Shiohira et al. (25)	2017	77	F	IAS	20	Unilocular, grayish-white jellylike, fluid	Syncope	Atrioventricular block; ventricular fibrillation
Smer et al. (26)	2017	52	M	IAS	10	NA	Paroxysmal	Atrial fibrillation
Miwa et al. **(**27)	2017	36	M	IAS	30	Unilocular, cream-colored jellylike fluid	Chest discomfort	Atrioventricular block
Li et al. (28)	2020	17	M	LA	90	NA	Chest pain and dyspnea	NA
Fukada et al. (2)	2020	42	F	IAS	29	Whitish-yellow colored mucous fluid	Dyspnea	NA
Saad et al. (29)	2021	31	F	IAS	56	Yellow fluid	Chest pain and palpitations	Arrhythmia
Chen et al. (30)	2022	49	M	RV	36	A yellowish-white gelatinous fluid	Chest pain and distress	NA
Fukudome et al. (31)	2022	31	F	RA	16	Yellowish-white, viscous mucous material	Palpitations and shortness of breath	Atrial fibrillation
Luo et al. (32)	2022	47	M	RA	33	Dark red fluid	Dizziness	NA
Gonzalez et al. (33)	2023	30	F	IAS	47	Tan-white rubbery	Chest pain	First-degree atrioventricular block

F, female; M, male; LA, left atrium; RA, right atrium; LV, left ventricle; RV, right ventricle, IAS, interatrial septum; IVS, interventricular septum; PLSVC, persistent left superior vena cava; NA, not available; ASD, atrial septal defect; VSD, ventricular septal defect.

The mean age of onset of bronchogenic cysts is 39, ranging from newborns to 77 years, with a female-to-male ratio of 2:1. The cysts are usually described as round or oval-shaped, unilocular masses that are either milky white or yellowish in color. The mean maximum size is 31 mm (range 10–90 mm). In general, the cysts grow slowly in the absence of complications (34). The most frequent location of the cysts is the IAS, accounting for more than half of the reported cases (1). Although the majority may be asymptomatic, many may present with syncope, dyspnea, chest pain, or palpitations (35, 36), as well as symptoms related to compression of surrounding structures (10–12). Associated congenital defects reported include atrial septal defects, ventricular septal defects, and persistent left superior vena cava.

Our patient, who presented with a third-degree atrioventricular block, was initially assessed using echocardiography and this was later confirmed through multimodality imaging. Due to the limitations associated with cardiac mass biopsy in clinical practice, diagnosis, and treatment of cardiac masses remain extremely challenging. The clinical diagnosis mainly relies on the comprehensive evaluation of echocardiography, CT, MRI, and other imaging techniques (36). Compared with cardiac CT and MRI, echocardiography can qualitatively assess and localize the cyst, and analyze its impact on hemodynamics, which is important for diagnosis and treatment planning. In this case, TTE showed a well-demarcated hypoechoic ovoid mass on the atrial septum, and TEE showed a well-defined mass with a regular shape, suggesting the possibility of a benign mass. MCE showed cystic wall-like enhancement around the mass, without internal perfusion, suggesting the possibility of a cystic lesion, and CT imaging also showed a well-defined non-enhancing spheroid or ovoid thin-walled lesion. MRI confirmed the absence of enhancement and intralesional fat, suggesting that the mass occupying the atrial septum was more likely to be benign. The characteristics of the mass seen on multimodality imaging were indicative of a benign atrial septal cystic mass. In addition, the patient’s atrioventricular block was due to the mass’s impact on myocardial electrophysiology, providing strong evidence for clinical decision-making. This was also confirmed after surgery.

Here, we presented a rare case of new-onset third-degree atrioventricular block that was initially found using echocardiography and subsequently confirmed by TEE, MCE, CT, and MRI. We also reviewed the imaging findings and highlighted the characteristic features of IBCs on multimodal imaging that are essential for appropriate diagnosis and management.

## Data Availability

The original contributions presented in the study are included in the article/Supplementary Material, further inquiries can be directed to the corresponding author.
